# Colonic Alimentation

**Published:** 1905-09

**Authors:** 


### Abstracts and Selections.

### Colonic Alimentation.

Dr. Jno. A. Wyeth read a paper before the Clinical Society of the New York Polyclinic on "Esophagotomy for the Removal of False Teeth" (*Journal A. M. A.*, July, 1905), and appended the following comments on colonic alimentation:

*Colonic Alimentation.*—The value of alimentation by the colon as a feature of surgical practice is so important that the following is submitted:

For conveying water by the lower bowel to the blood, normal salt solution should be employed (one teaspoonful of salt to one pint of water which has been boiled and allowed to cool down to the temperature of the body). From six to eight ounces to as much as a pint, and at times more than this, may be employed if absolutely necessary. The foot of the bed should be well elevated so that the liquid will gravitate away from the rectum and thus avoid pressure which induces an effort at evacuation. When there is great intolerance of the lower bowel smaller quantities should be employed with more frequent repetition.

*Milk Prepared by the Warm Process.*—One of the most useful foods given by the colon is milk. When the bowel is irritable the warm process, which is as follows, should be employed: Put a teacupful (gill) of cold water and the powder contained in one of the peptonizing tubes* into a clean quart bottle and shake thoroughly; add a pint of cold fresh milk and shake again; then place the bottle in a pail or kettle of warm water—about 115 F.—or not too hot to immerse the whole hand in without discomfort. Keep the bottle in the water bath for from thirty to forty minutes or longer if a greater degree of pre-digestion seems necessary, then put it immediately on ice. As a portion is needed, shake the bottle, pour out the quantity—usually four ounces—and heat gently to blood warmth. Avoid hasty heating and over-heating.

Six, eight, or twelve ounces may be given every four or five hours, or a larger quantity if the case is urgently in need of nutrition and the bowel is tolerant. In rectal feeding it is of great importance not to overcrowd the colon sufficiently to produce irritation.

*Cold Process.*—In cases where the enema can be retained for some time without irritation, the milk may be peptonized by the cold process. Put a teacupful (gill) of cold water into a clean quart bottle and dissolve it by shaking thoroughly the powder contained in one of the peptonizing tubes; add a pint of cold fresh milk, shake the bottle again and immediately place it directly in contact with the ice.

Warm each portion as it is required for injection, being careful to avoid hasty heating or over-heating.

---

*I prefer those prepared by Fairchild Brothers & Foster.

Or, only a sufficient quantity to use may be prepared each time by the following method: In two tablespoonfuls (one ounce) of cold water dissolve one-quarter of the contents of a peptonizing tube; add eight tablespoonfuls (four ounces) of cold milk; warm to the proper temperature and inject at once.

*Eggs.*—In administering eggs the following formula is advisable: Dissolve the white of an egg in three times its bulk of warm water; to this add the contents of one of the peptonizing tubes, stir well, and inject at once. The water should be just warm enough to make the mixture the proper temperature for the injection, not hot enough to coagulate the albumin.

Another method of using the whole of the egg is to beat the white and the yellow well together, with a pint of milk, adding a gill of water in which the contents of a peptonizing tube has been dissolved. It may be employed cold, but if it is thought best to use the warm method, warm water can be used as in the formula immediately preceding.

*Beef Juice.*—In using beef for rectal feeding, to a tablespoonful of minced lean beef add four tablespoonsful of cold water, and gradually heat to boiling. Strain all through a fine sieve or colander. When it becomes lukewarm add the contents of one of the peptonizing tubes, and it is ready for injection. More water may be added should it seem desirable.

*Peptonized Food and Whey.*—I have used Fairchild's panopepton in rectal feeding with much satisfaction. It should be diluted in two or three parts of lukewarm water, or preferably, normal salt solution.

If it be desired to employ whey, it can be combined with panopepton at times as follows: Put one pint of cold fresh milk in a clean saucepan and heat it to not over 100 F. Add two tablespoonsful of essence of pepsin and stir just enough to mix. Let it stand until firmly jellied, then beat with a fork until it is finely divided, and strain. Warm to the proper temperature and inject without dilution. Panopepton and whey may be used in conjunction by adding three parts of whey to one of panopepton.

Panopepton and peptonized milk may be used by mixing one tablespoonful of panopepton with two or three of peptonized milk prepared by the warm process. Mix the panopepton and peptonized milk when required for use.

